# Added value of the tumor-stroma ratio to the PREDICT model for breast cancer

**DOI:** 10.1016/j.breast.2026.104851

**Published:** 2026-06-24

**Authors:** Sophie C. Hagenaars, Tom A. Hueting, Frederike Dieleman, Judith R. Kroep, Ewout W. Steyerberg, Emad A. Rakha, Rob A.E.M. Tollenaar, Hein Putter, Andrew R. Green, Wilma E. Mesker

**Affiliations:** a Department of Surgery, Leiden University Medical Center, Leiden, the Netherlands; b Evidencio, Medical Decision Support, Haaksbergen, the Netherlands; c Department of Medical Oncology, Leiden University Medical Center, Leiden, the Netherlands; d Department of Biomedical Data Sciences, Leiden University Medical Center, Leiden, the Netherlands; e Nottingham Breast Cancer Research Centre, Academic Unit for Translational Medical Sciences, School of Medicine, The University of Nottingham, Nottingham City Hospital, Nottingham, United Kingdom

**Keywords:** Breast cancer, Tumor-stroma ratio, Prediction model, Prognosis, Histology

## Abstract

**Purpose:**

The tumor-stroma ratio (TSR) has repeatedly proven to be a strong prognostic and predictive marker in breast cancer. However, clinical integration of the TSR to further improve patient selection for breast cancer treatment and clinical decision making remains to be defined. Therefore, we evaluated the added value of the TSR to the well-recognized PREDICT model for survival outcomes following adjuvant systemic therapy.

**Methods:**

In total, 1770 patients from the Nottingham Breast Cancer Series were included. The incremental value of the TSR following addition to the PREDICT model regarding change in overall prediction model performance was evaluated based on Cox regression analyses and the area under the curve (AUC) for 5-, 10- and 15-year overall survival. Analyses were reported for the total patient group and stratified by molecular subgroups.

**Results:**

The largest model performance improvement was observed in the triple-negative breast cancer (TNBC) subgroup. At 5 years, the AUC ameliorated from 63.9 (95% confidence interval 55.1-72.7) to 70.9 (63.4-78.3) (p = 0.05) with a hazard ratio (HR) of 2.58 (1.39-4.79, p = 0.003). For 10-year survival, the HR was 2.02 (1.21-3.39, p = 0.007), with a rise in the AUC from 64.0 (55.6-72.3) to 69.0 (61.3-76.6) (p = 0.1). At 15 years after clinical diagnosis, the AUC increased from 64.6 (54.0-75.2) to 71.6 (61.1-82.1) at borderline significance (p = 0.06), together with an HR of 2.02 (1.24-3.27, p = 0.005).

**Conclusion:**

The AUC improved for the total patient cohort and all subgroups following the addition of the TSR, although the strongest and most significant model enhancements were observed in the TNBC subgroup.

## Highlights

• This study evaluates clinical implementation of the tumor-stroma ratio (TSR) as a histological biomarker for breast cancer.
• The goal is to improve clinical decision making in terms of patient selection for treatment and prognostication.
• Incremental values for 5-, 10- and 15-year overall survival were assessed after extending the PREDICT model with the TSR.
• Statistically significant model improvements in terms of the AUC were mainly observed for triple-negative breast cancer.
• The current study indicates that the TSR might aid in enhancing the identification of specific subgroups of breast cancer patients who are more responsive to certain breast cancer treatments and thereby further add to improving prognostication.

## List of abbreviations

AUC: Area Under the receiver operating characteristic Curve
CI: Confidence Interval
ER: Estrogen Receptor
H&E: Hematoxylin and Eosin
HER2: Human Epidermal growth factor Receptor 2
HR: Hazard Ratio
IQR: Interquartile Range
NPI+: Nottingham Prognostic Index Plus
PR: Progesterone Receptor
TNBC: Triple-Negative Breast Cancer
TSR: Tumor-Stroma Ratio

## 1 Introduction

The tumor-stroma ratio (TSR) is a histological parameter based on the relative amount of intratumoral stroma compared to tumor cells [1,2]. Over the last years, the TSR has been validated extensively in a large number of study populations as a predictive and prognostic parameter in breast cancer [[3], [4], [5], [6], [7], [8], [9], [10]], especially in triple-negative breast cancer (TNBC) [11]. Consequently, the next step would be to investigate its added value for clinical decision making, amongst others to clinical prediction models and the TNM classification [[12], [13], [14], [15]].

One of the prediction models that currently is ubiquitous in breast oncology clinical practice, is the PREDICT model [16,17]. This online tool can be used as a prognostic instrument to inform early-stage invasive breast cancer patients about 5-, 10- and 15-year survival rates and treatment benefits following different adjuvant systemic therapies. It considers several clinical parameters, including age at diagnosis, estrogen receptor (ER) status, human epidermal growth factor receptor 2 (HER2) status, invasive tumor size, tumor grade and the number of positive lymph nodes. Still, the model has limitations with regard to its predictive accuracy for 5-year survival of young women with ER-positive breast tumors [18] and slightly overestimates 10-year survival among older (aged ≥65) patients [19,20]. Besides, it does not include information regarding breast cancer histology, such as ductal or lobular breast cancer, or all currently available treatments, co-morbidity and lifestyle factors. Even though the overall discriminatory accuracy for survival prediction is high in several validation studies [[20], [21], [22], [23]], updating the PREDICT model with additional significant predictors could further improve the accuracy and thereby the selection of patients who will benefit from adjuvant treatment.

We aimed to evaluate the added value of the TSR to the PREDICT model in terms of 5-, 10- and 15-year survival accuracy following initial breast cancer diagnosis. Hereby, this will be the first study to assess the possibility of clinical implementation of the TSR as a histological prognostic and predictive parameter for breast cancer, aiming to improve clinical decision making in terms of patient selection for treatment and prognostication.

## 2 Methods

### 2.1 Study population

This study included data from the Nottingham Breast Cancer series, which consisted of retrospectively collected clinical data of 1794 women, who were all diagnosed with primary invasive breast cancer without distant metastases and were primarily treated with surgery in the Nottingham City Hospital (UK) between 1993 and 2002. Digital Hematoxylin and Eosin (H&E)-stained tissue slides were available for all patients. Detailed information has been reported previously [7].

Inclusion criteria were invasive breast carcinoma, followed by primary surgical treatment for all women between the age of 25 and 85. Patients were excluded in case of a medical history of breast cancer, previously administered neoadjuvant treatment and the presence of bilateral tumors. Moreover, in line with the PREDICT model, data had to be available for the variables age at diagnosis, invasive tumor size, number of positive lymph nodes, ER status, tumor grade and survival outcome. Lastly, treatment specifications per patient were necessary to optimally reflect the added value of the TSR to the PREDICT model, without the different therapies affecting survival outcomes.

Approval for the Nottingham Breast Cancer Series was obtained by the North West–Greater Manchester Central Research Ethics Committee under the title: Nottingham Health Science Biobank (NHSB), reference number 15/NW/0685. All patient data was anonymized and handled according to the national ethical guidelines (“Code for Proper Secondary Use of Human Tissue”, Dutch Federation of Medical Scientific Societies).

### 2.2 Tumor-stroma ratio

With regard to the Nottingham Breast Cancer Series, the TSR was previously assessed for the entire patient cohort with a Cohen's kappa in the total cohort of 0.87 (almost perfect level of agreement) [7]. In short, all digital H&E-stained slides were evaluated via CaseViewer software version 2.3 for Windows (3DHISTECH Ltd., Budapest, Hungary), following the method of Mesker et al. [2,24]. The whole tissue slide was visually assessed to select the area with the highest stroma abundance. After that, the amount of intratumoral stroma was independently scored per tenfold percentage by two trained observers, using a circular annotation with a predefined area of 3.1 mm^2^ [7,24]. A third observer was consulted if consensus could not be reached. Finally, tumors were categorized as either stroma-low (≤50% stroma) or stroma-high (>50% stroma), in line with previous TSR studies that have implemented the same cut-off level [1,2,7].

### 2.3 PREDICT model

Via Evidencio (https://www.evidencio.com), an open, online and freely accessible library for medical prediction models, access to the PREDICT model was obtained. Specifically for this study, the model ‘Survival breast cancer after surgery & bisphosphonates (PREDICT version 2.2)’ (model number 2313) was used. Patient-specific probability scores were computed by filling in all clinical data from the patients from the Nottingham Breast Cancer Series. Information was required on age at diagnosis, invasive tumor size, the number of positive lymph nodes, tumor grade, detection method (via screening or symptoms), ER status, HER2 status, Ki-67 status (positive in case Ki-67 > 10%), menopausal status, received treatments (chemotherapy, hormone therapy and/or Herceptin), and survival outcome in terms of overall survival (survival at 5, 10 and 15 years after diagnosis). Overall, in case of a missing value, the variable field could be entered as unknown, with the exception of age at diagnosis, invasive tumor size, number of positive nodes and survival outcome, which were obligatory in order to calculate a probability score.

### 2.4 Endpoints

The primary endpoint was to assess the incremental value of the TSR following addition to the PREDICT model for breast cancer in terms of the overall model performance using the entire patient cohort. Secondly, the added value was evaluated in the molecular subgroups (luminal A, luminal B, HER2-enriched and TNBC).

### 2.5 Statistical analysis

To evaluate the incremental value of the TSR following addition to the existing PREDICT model with regard to the change in overall model performance, the area under the receiver operating characteristic curve (AUC) was used [25,26]. Regarding this prediction performance parameter, increasing values resemble better model performance. Two Cox models were considered: the first model with PREDICT only, where the web-based probabilities, obtained using Evidencio's online PREDICT model, were converted to prognostic scores by calculating the complementary log-log transformation [27]; the second model with the PREDICT prognostic score and the TSR. The analyses were reported for the total group of patients and stratified by molecular subgroup (luminal A, luminal B, HER2-enriched and TNBC). All measures were calculated at 5, 10 and 15 years.

Patient characteristics were analyzed using IBM SPSS Statistics (version 25 for Windows). Continuous, non-normally distributed variables are presented as median (interquartile range; IQR) and categorical variables are shown as absolute numbers (percentage). Missing values for endocrine therapy (yes or no) and chemotherapy (no chemotherapy, 2nd generation chemotherapy or 3rd generation chemotherapy) were imputed using multiple imputation by chained equations (MICS) for the Nottingham Breast Cancer Series patient database.

Statistical analyses assessing the value of the TSR in addition to the PREDICT model scores were performed in R (version 4.4.0). The additional effect of the TSR to the PREDICT model scores was evaluated using multivariable Cox regression analyses, combining results on imputed data sets using Rubin's rules (package mitools, version 2.4 [28]), reported as hazard ratios (HRs), together with the 95% confidence intervals (CIs) and p-values. Statistical analyses to assess increments in prediction model performance in terms of the AUC at 5, 10 and 15 years after diagnosis were performed using package riskRegression, version 2023.12.21 [29]. For these analyses, the first imputed data asset was used. A p-value of less than 0.05 was considered statistically significant.

## 3 Results

### 3.1 Patient characteristics

Clinicopathological baseline characteristics of the Nottingham Breast Cancer Series patient cohort are shown in Table 1.

**Table 1:** Patient characteristics of the Nottingham Breast Cancer Series.

	Total patient group (n = 1770)	Stroma-high (n = 1100)	Stroma-low (n = 670)
**Age at diagnosis, years**	55 (48-63)	56 (48-63)	54 (47-62)
**Menopause**			
Yes	1249 (70.6%)	797 (72.5%)	452 (67.5%)
No	521 (29.4%)	303 (27.5%)	218 (32.5%)
**ER status**			
Positive	1459 (82.4%)	931 (84.6%)	528 (78.8%)
Negative	311 (17.6%)	169 (15.4%)	142 (21.2%)
**PR status**			
Positive	1064 (60.1%)	675 (61.4%)	389 (58.1%)
Negative	706 (39.9%)	425 (38.6%)	281 (41.9%)
**HER2 status**			
Positive	214 (12.1%)	130 (11.8%)	84 (12.5%)
Negative	1556 (87.9%)	970 (88.2%)	586 (87.5%)
**Ki-67 status**			
Positive	245 (13.8%)	130 (11.8%)	115 (17.2%)
Negative	83 (4.7%)	53 (4.8%)	30 (4.5%)
Missing	1442 (81.5%)	917 (83.4%)	525 (78.4%)
**Invasive tumor size, mm**	17 (12-23.25)	18 (14-25)	14 (10-21)
**Tumor grade**			
Grade 1	278 (15.7%)	174 (15.8%)	104 (15.5%)
Grade 2	730 (41.2%)	459 (41.7%)	271 (40.4%)
Grade 3	762 (43.1%)	467 (42.5%)	295 (44.0%)
**Method of detection**			
Screening	0 (0.0%)	0 (0.0%)	0 (0.0%)
Symptomatic	0 (0.0%)	0 (0.0%)	0 (0.0%)
Unknown	1770 (100.0%)	1100 (100.0%)	670 (100.0%)
**Lymph nodes**			
Positive	655 (37.0%)	431 (39.2%)	224 (33.4%)
Negative	1115 (63.0%)	669 (60.8%)	446 (66.6%)

Abbreviations: ER: estrogen receptor; PR: progesterone receptor; HER2: human epidermal growth factor receptor 2.

**Fig. 1:**
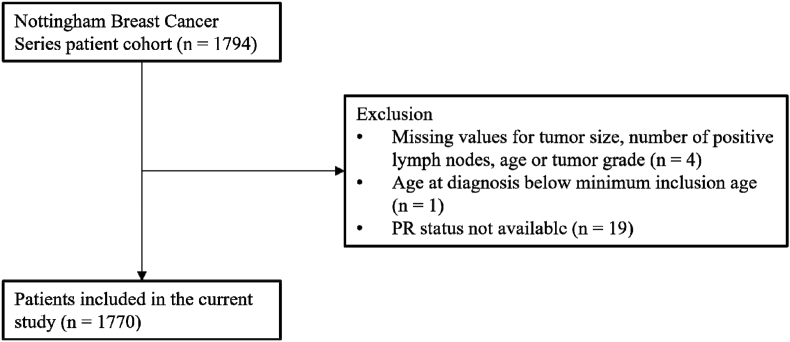
Consort diagram.

Out of the 1794 patients, a total of 1770 patients were evaluated in the current study (Fig. 1). Five patients were excluded due to missing values for either tumor size, number of positive lymph nodes, age, or tumor grade (n = 4), and because of an age at diagnosis of 23 years (n = 1), which was below the minimum inclusion age according to the specifications of the PREDICT model. Additionally, progesterone receptor (PR) status was not available for 19 patients, and therefore, these patients could not be evaluated in the molecular subgroup analyses. After that, the study group consisted of women with a median age at time of diagnosis of 55 years (IQR 48-63). Overall, 1100 (62.1%) patients had stroma-high tumors and 670 (37.9%) tumors were considered stroma-low.

### 3.2 Nottingham Breast Cancer Series

Results for the Nottingham Breast Cancer Series are both shown for the entire patient cohort (n = 1770; Table 2), and stratified by molecular subgroup (Table 3): luminal A (n = 991), luminal B (n = 468), HER2-enriched (n = 76) and TNBC (n = 235).

**Table 2:** Results from the total patient cohort for 5-, 10- and 15-year survival: incremental value of the TSR following addition to the PREDICT model in terms of overall model performance (AUC).

	5-year survival	10-year survival	15-year survival
**PREDICT only**	75.2 (71.5-78.9)	68.4 (65.2-71.6)	69.7 (65.6-73.8)
**PREDICT + TSR**	75.6 (71.9-79.3)	68.7 (65.6-71.9)	69.8 (65.7-73.9)

AUC is shown as AUC value and 95% CI. Abbreviations: TSR: tumor-stroma ratio; AUC: area under the receiver operating characteristic curve; CI: confidence interval.

**Table 3:** Molecular subgroup analyses for 5-, 10- and 15-year survival: incremental value of the TSR following addition to the PREDICT model in terms of overall model performance (AUC).

	5-year survival		10-year survival		15-year survival	
**Luminal A**						
PREDICT only	67.8 (59.8-75.8)		67.1 (61.9-72.3)		69.6 (63.8-75.4)	
PREDICT + TSR	68.6 (60.8-76.3)		68.5 (63.4-73.6)		69.5 (63.7-75.2)	
**Luminal B**						
PREDICT only	72.2 (65.8-78.7)		65.3 (59.7-70.9)		70.6 (62.7-78.6)	
PREDICT + TSR	72.3 (65.9-78.8)		65.5 (59.9-71.2)		70.9 (63.1-78.7)	
**HER2-enriched**						
PREDICT only	86.4 (78.1-94.6)		76.9 (65.2-88.7)		60.9 (36.0-85.7)	
PREDICT + TSR	86.6 (78.3-94.9)		77.1 (65.5-88.8)		67.6 (43.4-91.9)	
**TNBC**						
PREDICT only	63.9 (55.1-72.7)		64.0 (55.6-72.3)		64.6 (54.0-75.2)	
PREDICT + TSR	70.9 (63.4-78.3)		69.0 (61.3-76.6)		71.6 (61.1-82.1)	

AUC is shown as AUC value and 95% CI. Abbreviations: TSR: tumor-stroma ratio; TNBC: triple-negative breast cancer; AUC: area under the receiver operating characteristic curve; CI: confidence interval.

#### 3.2.1 Total patient cohort

First, for the 5-year survival (n = 187 events), the TSR had a significant additional value to the PREDICT model, depicting a worse survival for stroma-high patients compared to patients with stroma-low tumors (HR 1.39, 95% CI 1.02-1.89, p = 0.037). The AUC increased from 75.2 (95% CI 71.5-78.9; PREDICT only) to 75.6 (95% CI 71.9-79.3) when the TSR values were added to the PREDICT model (p = 0.2). After 10 years, the number of events was 355, and the HR of the TSR addition was 1.24 at a borderline significant level (95% CI 0.99-1.55, p = 0.059). The AUC went from 68.4 (95% CI 65.2-71.6) to 68.7 (95% CI 65.6-71.9) (p = 0.2). Lastly, at 15 years after initial clinical diagnosis, 462 events had occurred. Analyses revealed an HR of 1.12 for the addition of the TSR to the PREDICT model (95% CI 0.93-1.36, p = 0.24). For this survival period, the model showed a minimal AUC increase from 69.7 (95% CI 65.6-73.8) to 69.8 (95% CI 65.7-73.9) with a p-value of 0.7. An overview of these results for the total patient cohort is shown in Table 2.

#### 3.2.2 Luminal A subgroup

At 5 years, the number of events was 50 and the HR for the TSR addition 1.69 (95% CI 0.86-3.33, p = 0.13). The AUC showed a rise in value (67.8 [95% CI 59.8-75.8] to 68.6 [95% CI 60.8-76.3]; p = 0.6). Comparable results were found for the 10-year survival accuracy (n = 139 events): the AUC metric went from 67.1 (95% CI 61.9-72.3) to 68.5 (95% CI 63.4-73.6) (p = 0.2). Meanwhile, adding the TSR to the model showed to have a statistically significant effect at an HR of 1.57 (95% CI 1.06-2.32, p = 0.023). Lastly, the positive effect of the TSR addition to the PREDICT model was still visible for the 15-year survival predictions after which 207 events had occurred (69.6 [95% CI 63.8-75.4] to 69.5 [95% CI 63.7-75.2], p = 0.8), although the added value of the TSR was no longer statistically significant (HR 1.23, 95% CI 0.91-1.66, p = 0.18).

#### 3.2.3 Luminal B subgroup

In the luminal B subgroup, the overall added value of the TSR to the PREDICT model in terms of the increase in AUC was very minor. For the 5-year survival (n = 64 events), there was no significant difference between the AUCs: 72.2 (95% CI 65.8-78.7) to 72.3 (95% CI 65.9-78.8) with a p-value of 0.5. Additionally, the Cox model results following the addition of the TSR were not statistically significant (HR 1.04, 95% CI 0.62-1.75, p = 0.88). There was likewise only a small, statistically insignificant increase in model performance for the 10-year overall survival (n = 121 events; AUC from 65.3 [95% CI 59.7-70.9] to 65.5 [95% CI 59.9-71.2] with p = 0.7; while the HR of the TSR lowered [HR 0.87, 95% CI 0.61-1.26, p = 0.47]), or at 15-year survival (n = 147 events; AUC from 70.6 [95% CI 62.7-78.6] to 70.9 [95% CI 63.1-78.7] with a p-value of 0.8) together with the Cox analyses revealing an HR of 0.85 for the addition of the TSR to the PREDICT model (95% CI 0.61-1.18, p-value = 0.34).

#### 3.2.4 HER2-enriched subgroup

For the 5-year survival, 22 events were observed and a HR of 0.70 was found for the addition of the TSR to the PREDICT model (95% CI 0.29-1.70, p = 0.43), while the AUC increased from 86.4 (95% CI 78.1-94.6) to 86.6 (95% CI 78.3-94.9) at a p-value of 0.7. Model improvement for the 10-year survival period (n = 28 events) was similar to the 5-year survival results with only a minor AUC improvement: 77.1 (95% CI 65.5-88.8, p = 0.9), compared to 76.9 (95% CI 65.2-88.7) for the PREDICT-only model performance. The effect of the addition of the TSR to the Cox model with the PREDICT scores led to an HR of 0.73 (95% CI 0.33-1.59) together with a p-value of 0.42. After 15 years, there were 32 events, and the model performance showed the strongest, significant improvement within the HER2-enriched subgroup, namely from 60.9 (95% CI 36.0-85.7) to 67.6 (95% CI 43.4-91.9) with regard to the AUC metric (p = 0.02). The HR lowered to 0.58 (95% CI 0.28-1.20), but was not at a statistically significant level (p = 0.14).

#### 3.2.5 TNBC subgroup

Five-year survival prediction accuracy (n = 51 events) ameliorated at a significant level from 63.9 (95% CI 55.1-72.7) to 70.9 (95% CI 63.4-78.3) in terms of the AUC (p = 0.05), next to the significant effect of the TSR addition to the Cox model (HR 2.58, 95% CI 1.39-4.79, p = 0.0028). A similar model improvement was observed at 10 years, at which time 67 events were observed: the addition of the TSR revealed an HR of 2.02 (95% CI 1.21-3.39, p = 0.0074) and the AUC showed a rise (64.0 [95% CI 55.6-72.3] to 69.0 [95% CI 61.3-76.6]; p = 0.1). Finally, for the 15-year survival (n = 76 events), the Cox analysis showed an HR of 2.02 for the TSR (95% CI 1.24-3.27, p = 0.0046) and a positive development after adding the TSR to the PREDICT model (AUC from 64.6 [95% CI 54.0-75.2] to 71.6 [95% CI 61.1-82.1]), at a borderline significant level (p = 0.06).

## 4 Discussion

In this study, the added value of the TSR to the currently existing PREDICT model for breast cancer prognosis following treatment was evaluated in terms of change in overall model performance. Analyses were performed for the total group of patients and split by molecular subgroups for 5-, 10- and 15-year overall survival. The AUC improved for all groups (both the total patient cohort and the molecular subgroups) following the addition of the TSR, although the strongest and most significant model enhancements were observed in the TNBC subgroup.

This is the first study in which a possible mode for clinical implementation of the TSR in daily pathology diagnostics as a histological parameter for breast cancer was assessed by adding it to a regularly used clinical prediction model for treatment benefits following adjuvant treatment. Although the prognostic effect has repeatedly been demonstrated by various study groups [11,30] and the additional costs and time for clinical assessment are expected to be low, the optimal route to clinical implementation still has yet to be discovered. Here, the PREDICT tool, an online clinical decision-making tool, was evaluated, but other breast cancer prediction models could be considered in the future as well, such as the PORTRET tool to predict recurrence, overall survival and other causes of mortality among older breast cancer patients [31] and the Nottingham Prognostic Index Plus (NPI+) [32,33].

Although incremental values in overall prediction model performance were observed for the total group of patients and all subgroups, the largest and most statistically significant effects were noted in the TNBC subgroup. This result was not unexpected, since the TSR has repeatedly shown to have the strongest prognostic and predictive effects in TNBC [1,3,5,7,11,30]. As this molecular subgroup shows to have relatively high tumor aggressiveness, early tumor recurrence and high mortality compared to other breast cancer tumors [34], improvements in patient stratification for optimal treatment regimens are clinically most relevant and should be subject for further studies. An interesting future study perspective would be whether certain histological biomarkers, such as the TSR, could be valuable for patient selection for neoadjuvant immunotherapy in this subgroup, because these new therapies have shown very promising results regarding complete pathological response for a large part of the TNBC patients [[35], [36], [37], [38], [39]].

Another notable outcome is that the TSR shows less statistically significant correlations with survival, especially in the total group of patients, as compared to previous studies in which the effect of stroma-high tumors with a shorter survival were stronger [7,11,40]. This can partly be explained by the fact that the overall survival was used in this study, in line with the PREDICT model, instead of the more commonly used recurrence-free survival or breast-cancer specific survival. Therefore, especially for the longer follow-up times, other causes of death could have interfered with our current outcome. Moreover, here, the TSR was added to the existing PREDICT model, which includes many parameters that the TSR has also shown to be correlated to, such as tumor grade and age [7,41]. This might contribute to the smaller effects in the current study. Lastly, in the Luminal B and HER2-enriched subgroups, the addition of the TSR to the PREDICT model even led to HRs that were in favor of stroma-high tumors, although none of these results were statistically significant.

The TSR is currently being assessed by visual eyeballing using H&E-stained slides, scored per tenfold percentage by two independent observers and split into the stroma-high and stroma-low groups based on the cut-off value of 50% stroma [24]. Although the Cohen's kappa values for interobserver agreement are generally high [11,24,42], the level of subjectivity could be reduced, the reproducibility improved and the borderline cases around the 50% cut-off point more accurately classified, if scoring would be performed using image analysis and artificial intelligence. A number of studies have already been performed to analyze possible modes for automation: for instance the use of polarized light microscopy [43] and digital image analysis performed on tissue microarrays [9,44] in breast cancer, and automated analyses in user-provided stroma hot-spots using deep learning in rectal cancer [45]. Using a convolutional neural network to score whole-slide images in colorectal [46] and breast [8] cancer has proven successful and will open avenue for automated assessment of the TSR in routine practice. Although these tools have not yet been validated externally or are currently still under investigation, the existing data provide evidence of their potential clinical relevance. Therefore, developing and validating a robust method for objective, reproducible TSR scoring will be the next step in the process of speeding up implementation in daily diagnostics and uptake into the clinic.

Although improvements of the AUC were seen for the total study population and for all subgroups for 5-, 10- and 15-year overall survival, which were essentially only significant in the TNBC subgroup, as opposed to most other subgroups, it remains difficult to depict which incremental values for this performance measure can be defined as substantial following the addition of the histological TSR marker to the currently existing PREDICT model [25]. For instance, increase in AUC can be small if the baseline value was already high [47,48]. For this reason, interpretation of the present increases in AUC and its relevance for clinical practice is challenging, although any model improvement that leads to optimalization of predicting 5-, 10- and 15-year survival outcomes following different adjuvant treatment regimens might be beneficial to optimize patient selection for treatment, especially in case of a relatively easily implementable marker such as the TSR. Therefore, other factors, such as cost-effectiveness, should also be considered in the process of adopting a novel marker in an already existing prediction model. Additionally, in studies in which a conventional prediction model is extended with an additional biomarker, there is a risk of overoptimistic expectations of how the marker will perform. Therefore, the incremental value (i.e. model performance without versus with the new marker) should be assessed in independent, external data [26], as was done in this study using the Nottingham Breast Cancer Series. Notably, this series comprises only a single cohort, so for the current increase in incremental value to be considered robust and for it to be implemented in the PREDICT model, another independent validation with a second cohort will ultimately be required. Lastly, there was a tendency for the added effect of the TSR in terms of the HR to become smaller over time (i.e. non-proportional hazards); the effect of the 5-year analysis was higher than that of the 15-year analysis. Therefore, it seems that, especially for studies with a short follow-up, it is even more worthwhile to add the TSR to the PREDICT model.

This study has a few limitations. First, with regard to the choice for the PREDICT model as a possible route for clinical implementation of the TSR parameter, this prediction model does not include all currently available treatments (e.g. radiotherapy), it does not differentiate between the extent of the surgery (i.e. lumpectomy or mastectomy), it does not consider tumor histology and it does not take lifestyle factors into account. Therefore, the clinical value and representativeness of the current standard for breast cancer treatment should be evaluated. Additionally, the model does not consider the possibility of neoadjuvant treatment, while this is increasingly prescribed for early breast cancer, especially neoadjuvant chemotherapy in case of HER2-positive tumors or TNBC [49]. Fortunately, regarding the assessment of the TSR, this would not be a limitation, as the TSR can both be determined on tumor biopsies and resection specimen [[50], [51], [52]]. Therefore, if neoadjuvant treatment will one day become part of the PREDICT model, the TSR can contribute to patient selection for treatment, as has been shown for breast cancer [5,53,54] and other tumor types [[55], [56], [57], [58]]. Lastly, regarding the patient database that was used in this study, a retrospective cohort was assessed in which the follow-up period had to be minimally 15 years in order to study model improvement for the 15-year survival period, next to the 5- and 10-year survival periods. However, whilst all patients who were evaluated in the cohort were treated with similar techniques and therapies, the number of possible treatment strategies have more recently increased, which might affect survival outcomes.

In conclusion, in this study, the incremental values following the extension of the PREDICT model for breast cancer with the histological TSR parameter were evaluated in terms of changes in prediction model performance for 5-, 10- and 15-year overall survival. Mainly for the TNBC subgroup, for which improvements in patient stratification are clinically most relevant due to its high tumor aggressiveness, statistically significant model improvements in terms of the AUC were observed. Although the outcomes were less significant in the other groups regarding the AUC, all analyses did show improvements for the AUC. Additionally, for the total group of patients, the TNBC subgroup and the Luminal A subgroup, the HR for overall survival following the addition of the TSR to the PREDICT model remained in favor of stroma-low tumors, at significant levels for most of the outcomes. Therefore, adding the TSR to the PREDICT model can be considered, especially for the TNBC subgroup. Moreover, the assessment of the histological TSR parameter has low costs and takes little extra time, lowering the threshold to implement it in daily pathology practice. Hereby, the TSR can aid in enhancing the identification of specific subgroups of breast cancer patients who are more responsive to certain breast cancer treatments and thereby further add to prognostication.

## Ethics approval

Approval for the Nottingham Breast Cancer Series was obtained by the North West–Greater Manchester Central Research Ethics Committee under the title: Nottingham Health Science Biobank (NHSB), reference number 15/NW/0685. All patient data was anonymized and handled according to the national ethical guidelines (“Code for Proper Secondary Use of Human Tissue”, Dutch Federation of Medical Scientific Societies).

## Data availability statement

The datasets analyzed during our study are available from the corresponding author on reasonable request. The prediction model is available for external validation via Evidencio (https://www.evidencio.com; model ID number 2313).

## Funding

This work was supported by grants from the Bollenstreekfonds, Lisse, the Netherlands. No grant number applicable.

## CRediT authorship contribution statement

**Sophie C. Hagenaars:** Conceptualization, Data curation, Formal analysis, Investigation, Methodology, Project administration, Validation, Writing – original draft, Writing – review & editing. **Tom A. Hueting:** Conceptualization, Formal analysis, Methodology, Software, Writing – review & editing. **Frederike Dieleman:** Formal analysis, Writing – review & editing. **Judith R. Kroep:** Supervision, Writing – review & editing. **Ewout W. Steyerberg:** Methodology, Supervision, Writing – review & editing. **Emad A. Rakha:** Data curation, Project administration, Resources, Supervision, Writing – review & editing. **Rob A.E.M. Tollenaar:** Supervision, Writing – review & editing. **Hein Putter:** Formal analysis, Methodology, Supervision, Validation, Writing – review & editing. **Andrew R. Green:** Data curation, Project administration, Resources, Supervision, Writing – review & editing. **Wilma E. Mesker:** Conceptualization, Data curation, Funding acquisition, Methodology, Project administration, Supervision, Writing – review & editing.

## Declaration of competing interest

The authors declare the following financial interests/personal relationships which may be considered as potential competing interests: Dr. W.E. Mesker reports financial support was provided by Bollenstreekfonds, Lisse, the Netherlands (no grant number applicable). Dr. T.A. Hueting reports a relationship with Evidencio that includes: board membership. The other authors have no known competing financial interests or personal relationships that could have appeared to influence the work reported in this paper.
